# rIL-10 enhances IL-10 signalling proteins in foetal alveolar type II cells exposed to hyperoxia

**DOI:** 10.1111/jcmm.12596

**Published:** 2015-06-08

**Authors:** Hyeon-Soo Lee, Dong Gun Lee

**Affiliations:** a Department of Pediatrics, Dongtan Jeil Women and Infants’ HospitalWhasung, South Korea; b Institute of Medical Sciences, Kangwon National University School of MedicineChuncheon, Kangwon, South Korea; c Medical and Bio-Materials Research Center, Kangwon National University School of MedicineChuncheon, Kangwon, South Korea

**Keywords:** IL-10 signalling proteins, IL-10 receptors, hyperoxia, foetal alveolar type II cells, IL-8

## Abstract

Although the mechanisms by which hyperoxia promotes bronchopulmonary dysplasia are not fully defined, the inability to maintain optimal interleukin (IL)-10 levels in response to injury secondary to hyperoxia seems to play an important role. We previously defined that hyperoxia decreased IL-10 production and pre-treatment with recombinant IL-10 (rIL-10) protected these cells from injury. The objectives of these studies were to investigate the responses of IL-10 receptors (IL-10Rs) and IL-10 signalling proteins (IL-10SPs) in hyperoxic foetal alveolar type II cells (FATIICs) with and without rIL-10. FATIICs were isolated on embryonic day 19 and exposed to 65%-oxygen for 24 hrs. Cells in room air were used as controls. IL-10Rs protein and mRNA were analysed by ELISA and qRT-PCR, respectively. IL-10SPs were assessed by Western blot using phospho-specific antibodies. IL-10Rs protein and mRNA increased significantly in FATIICs during hyperoxia, but JAK1 and TYK2 phosphorylation showed the opposite pattern. To evaluate the impact of IL-8 (shown previously to be increased) and the role of IL-10Rs, IL-10SPs were reanalysed in IL-8-added normoxic cells and in the IL-10Rs’ siRNA-treated hyperoxic cells. The IL-10Rs’ siRNA-treated hyperoxic cells and IL-8-added normoxic cells showed the same pattern in IL10SPs with the hyproxic cells. And pre-treatment with rIL-10 prior to hyperoxia exposure increased phosphorylated IL-10SPs, compared to the rIL-10-untreated hyperoxic cells. These studies suggest that JAK1 and TYK2 were significantly suppressed during hyperoxia, where IL-8 may play a role, and rIL-10 may have an effect on reverting the suppressed JAK1 and TYK2 in FATIICs exposed to hyperoxia.

## Introduction

Bronchopulmonary dysplasia (BPD) is the most common cause of morbidity and mortality in preterm infants 1. Bronchopulmonary dysplasia has a multifactorial aetiology, but one of the most immediate and frequent causes of BPD is lung injury imposed by hyperoxia 1,2.

Nowadays, a growing amount of attention has been paid to ‘new’ BPD distinguished from ‘old’ BPD. ‘New’ BPD is clinically characterized by progressive deterioration in lung function following a few days of supplemental oxygen after birth 3, and the pathological findings of ‘new’ BPD are characterized by irreversible alveolar and capillary rarefaction 4 and alveolar simplification with disorganized vasculature 5 leading to developmental arrest of the lungs 6,7. Although the mechanisms of generating ‘new’ BPD are not yet clearly defined, these clinical and pathological characteristics of ‘new’ BPD may be related to the fact that the failure of alveolar type II cells to proliferate during the first postnatal week may permanently alter postnatal lung growth during a critical period of postnatal lung development 8. This could play an important role in the evolution of ‘new’ BPD from an early postnatal age in preterm infants 8. Previous observations from our laboratory also support this idea by showing that hyperoxia-induced type II cell injury including cellular necrosis begins from a very early stage of hyperoxia (<12 hrs) 9,10. Considering these evidence, it is highly suggested to pay closer attention to minimizing lung injury, especially type II cell injury starting in the early postnatal stage in preterm newborns. However, unfortunately, there are no specific measures for the protection of preterm lungs against injury induced from a very early stage (<6 hrs) of hyperoxia 9,10.

Recently, there is growing concern regarding the limited production of anti-inflammatory cytokine, interleukin (IL)-10 which regulates inflammation, as a factor in the development of BPD in preterm infants 11–13. A previous study reported that IL-10 production decreased in tracheal aspirates from infants with BPD 14. Moreover, another previous study showed that recombinant IL-10 (rIL-10) significantly inhibited IL-6 and TNFα production in macrophages from tracheal aspirate fluid from newborn infants 15. We also reported that an imbalance between pro- and anti-inflammatory cytokine, IL-10 is generated in FATIICs during hyperoxia, and pre-treatment of rIL-10 protected type II cells from injury induced from the early stage of hyperoxia 9.

Interleukin-10 is the most potent anti-inflammatory cytokine described to date 16. Interleukin-10 exerts its anti-inflammatory actions through binding to IL-10 receptors (α and β); this binding subsequently stimulates phosphorylation and activation of intracellular signalling pathways, specifically the JAK-STAT signalling pathway 17,18. A previous study showed that a lack of IL-10 receptor complexes did not have a direct effect on the expression of adhesion molecules and pro-inflammatory cytokines by endothelial cells 19. Another study showed that IL-10 had no anti-inflammatory action in macrophages in mice with disrupted genes for JAK1 and STAT3 20.

Based on our former investigations, we could speculate the therapeutic potency of IL-10 to prevent or attenuate lung injury induced from the very early stage of hyperoxia in preterm lungs. Thus, we investigated next the expression of IL-10 receptors and the activation of IL-10 signalling proteins in FATIICs exposed to hyperoxia and re-analysed them after pre-incubating FATIICs with rIL-10 prior to hyperoxia exposure, using an *in-vitro* model in which rat FATIICs were isolated on embryonic day 19 (E19) of gestation (transition from the canalicular to the sacular stage of lung development).

## Materials and methods

### Cell isolation, hyperoxia and treatment protocol

All animal work was approved by the Institutional Animal Care at the Kangwon National University. Foetal rat lungs were obtained from time-pregnant Sprague–Dawley rats (Daehan Biolink, Eumsung, South Korea) on E19 (term = 22 days). After extraction of foetal lungs and isolation of type II cells was proceeded as described previously 9,21,22 Describing briefly, extracted tissues were finely minced and digested with 0.5 mg/ml collagenase type I and 0.5 mg/ml collagenase type IA (Sigma Chemical Co., St. Louis, MO, USA) with vigorous pipetting for 15 min. at 37°C. After collagenase digestion, the suspension was centrifuged and, the pellet was resuspended in DMEM with 10% (vol/vol) and 20% foetal serum bovine serum and cell suspensions were sequentially filtered through 100-, 30- and 20-μm nylon meshes using screen cups (Sigma Chemical Co.). The filtrate from 20-μm nylon mesh, containing mostly fibroblasts, was discarded. Clumped non-filtered cells from the 30- and 20-μm nylon meshes were collected after several washes with DMEM to facilitate filtration of non-epithelial cells. Further type II cell purification was achieved by incubating cells in 75-cm^2^ flasks for 30 min. Non-adherent cells were collected and cultured overnight in 75-cm^2^ flasks containing serum-free DMEM. Purity of the type II cell fraction was determined to be 90 ± 5% by microscopic analysis of epithelial cell morphology and immune-blotting for cytokeratin/surfactant protein-C and vimentin as markers of epithelial cells and fibroblasts, respectively 23. After overnight culture, type II epithelial cells were harvested with 0.25% (wt/vol) trypsin in 0.4 mM ethylenediaminetetraacetic acid (EDTA) and plated at a density of 10 × 10^5^ cells/well on 6-well plates pre-coated with laminin (10 μg/ml). Plates containing adherent cells were maintained for an additional 24 hrs in serum-free DMEM and then incubated in a culture chamber with ProOx Oxygen Controller with Low profile right angle sensor (BioSpherix, Redfiled, NY, USA). 65%-hyperoxia was applied for 24 hrs, and cells grown in room air (5% CO_2_) were treated in an identical manner and served as controls. The incubation time was set at 24 hrs based on the evidence showing that alveolar type II cells had no obvious morphological changes after exposure to 95%-hyperoxia for 48 hrs 24, and the time of transformation from the alveolar type II cells to the type I cells in rat was about 2 days 25. In the experiments with the pre-incubation of rIL-10 (R&D Systems, Minneapolis, MN, USA), the cells cultured in an identical manner were treated with rIL-10 at a concentration of 250 ng/ml 9 for 1 hr before hyperoxia exposure. The concentration of rIL-10, 250 ng/ml was chosen based on our previous study showing that 250 ng/ml of rIL-10 affected greatly on reducing cell death and IL-8-release in foetal alveolar type II cells exposed to hyperoxia 9.

### ELISA for IL-10 and IL-10 receptors

After experiments, the supernatants were collected, and the cells were harvested. They were stored at −80°C prior to analysis. ELISA for IL-10 in the supernatants was performed according to the manufacturer’s instructions (Quantine, cat # R1000; R&D Systems). For the analysis of receptors, samples were homogenized in ice-cold Tris-50 mM/EDTA-1 mM buffer containing a cocktail of protease (Roche, Mannheim, Germany) and phosphatase inhibitors (Sigma Chemical Co.) using a potter homogenizer. Interleukin-10 receptors concentrations were measured using ELISA kits according to the manufacturer’s instructions (IL-10 receptor-α: cat # E91626Rα; USCNK, Houston, TX, USA and IL-10 receptor-β: cat #: E91638Rβ; USCNK). Interleukin-10 receptor-α and -β were analysed in the cells treated with IL-8, 100 ng/ml (BioNEER, Daejeon, South Korea) with the identical manner. The dose of IL-8, 100 ng/ml, was decided based on our previous investigation, where IL-8 was increasingly released approximately by 100 ng/ml after 24 hrs of hyperoxia exposure, as compared to the normoxic cells at 24 hrs 9.

### qRT-PCR

Total RNA was extracted from E19 type II cells exposed to 65%-hyperoxia for 24 hrs or parallel normoxic samples by a single-step method and purified further with the Rneasy Mini Kit (Invitrogen, Carlsbad, CA, USA). Fold expressions of the hyperoxic samples relative to the controls were calculated using the ΔΔC_T_ method for relative quantification as described previously 9,21,22. Samples were normalized to the 18S rRNA. TaqMan primers from Assays-on-Demand™ Gene Expression Products (Applied Biosystems, Carlsbad, CA, USA), and the following primers were used: IL-10 (cat #: Rn99999017_ml; Invitrogen); IL-10 receptor-α (cat #: Rn00589389_m1; Invitrogen); IL-10 receptor-β (cat #: Rn01477368_m1; Invitrogen); and 18S (cat #: Hs99999901_s1; Invitrogen). 5 μg of total RNA were reverse-transcribed into cDNA by the Superscript Double Stranded cDNA Synthesis kit (Invitrogen). To amplify the cDNA by qRT-PCR, 5 μl of cDNA were added to a mixture of 25 μl of TaqMan Universal PCR Master Mix (Applied Biosystems) and 2.5 μl of 20× Assays-on-Demand™ Gene Expression Assay Mix containing forward and reverse primers and TaqMan-labelled probe (Applied Biosystems). The reactions were performed in an ABI Prism 7000 Sequence Detection System (Applied Biosystems).

### Western blot

Monolayers were lysed in RIPA buffer [20 mM Tris-HCl (pH 8.0), 137 mM NaCl, 1% Triton X-100, 10% glycerol, 2 mM EDTA, 1 mM sodium vanadate, 1 M β-glycerophosphate, 0.5 M sodium fluoride, 1 mM phenylmethylsulfonyl fluoride]. Lysates were centrifuged and total contents were determined using BCA protein assay kit. 50 μg of protein were separated by SDS-PAGE and transferred to polyvinylidene difluoride membranes. Blots were hybridized with polyclonal antibodies (p-JAK1: cat #: CE3331; Cell Signaling, Denvers, MA, USA; p-TYK2: cat #: SC-11763; Santa Cruz Biotechnology, Dallas, TX, USA; and p-STAT3: cat #: CE9131; Cell signaling) as described previously 9,21,22. Anti-rabbit Ig G was used as secondary antibody, and conjugated with horseradish peroxidase, and blots were developed by exposing them to X-ray film. Membranes were then stripped and reprobed with total antibodies (t-JAK1: cat #: CE3344s; Cell signaling; t-TYK2: cat #: SC-7205; Santa Cruz Biotechnology; and t-STAT3: cat #: CE9132s; Cell signaling) and actin antibody in an identical manner 9,21,22.

### Transfection with Small Interference RNA

Interleukin-10 receptor-α and -β specific siRNA and control siRNA were obtained from BioNEER. Foetal type II cells were grown to 70% confluence and transfected with 50 nM siRNA IL-10 receptor-α and -β using Lipofectamine Plus reagents (Invitrogen) according to the manufacturer’s instructions. The level of p-JAK1, p-TYK2 and p-STAT3 were analysed by Western blot at 24 hrs after transfection.

### Statistical analysis

Results are expressed as means ± SD from at least three experiments. anova was used to compare values for IL-8 and IL-10 between oxygen-concentrations, and the other values were analysed using unpaired Student’s *t*-test. *P* < 0.05 was considered statistically significant.

## Results

### IL-10 production decreases in an oxygen-concentration-dependent manner

**Figure 1:**
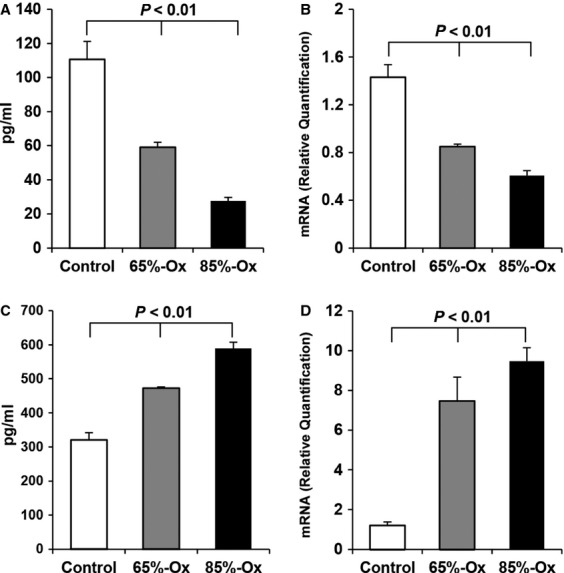
IL-10 and IL-8 during hyperoxia. E19 foetal alveolar type II cells were exposed to 65%-hyperoxia for 24 hrs. The released IL-10 and IL-8 in the supernatant and the genes expression of IL-10 and IL-8 were analysed by ELISA (A and C) and qRT-PCR (B and D), respectively. Graphical depiction showing that 65%-hyperoxia down-regulated IL-10 protein (A) and IL-10 mRNA (B) and up-regulated IL-8 protein (C) and IL-8 mRNA (D). The results are represented as the mean ± SD from three different experiments.

The released IL-10 in the supernatant and the gene expression of IL-10 were analysed by ELISA and qRT-PCR, respectively. The results show that the released IL-10 in FATIICs was significantly reduced by 41% and 74% after exposure to 65%- and 85%-hyperoxia for 24 hrs, respectively, compared to the controls (Fig.1A), and IL-10 mRNA had similar patterns as the ELISA findings (Fig.1B). In contrast, the released IL-8 and the expression of IL-8 mRNA showed the opposite pattern to IL-10 during hyperoxia (Fig.1C and D). The released IL-8 increased 1.5-fold and 1.7-fold after exposure to 65%- and 85%-hyperoxia for 24 hrs, respectively, as compared to the controls (Fig.1C), and IL-8 mRNA increased 6.2-fold and 7.8-fold after 65%- and 85%-hyperoxia for 24 hrs, respectively, compared to the controls (Fig.1D).

### IL-10 receptors increase in FATIICs during hyperoxia

**Figure 2:**
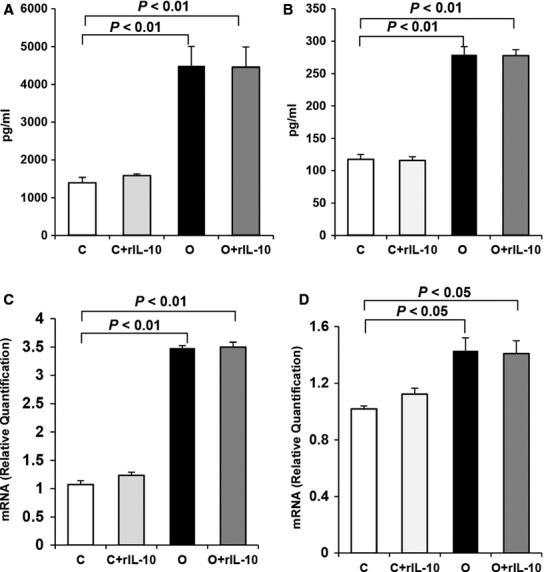
IL-10 receptors protein and gene expression in the hyperoxic cells and in the rIL-10-treated cells. E19 foetal alveolar type II cells were exposed to 65%-hyperoxia for 24 hrs. Expression of IL-10 receptors was assessed by ELISA (A and B) and qRT-PCR (C and D), respectively. Graphical depiction showing IL-10 receptor-α (A) and -β (B) proteins and IL-10 receptor-α mRNA (C) and -β mRNA (D) increased significantly in the hyperoxic and in the rIL-10-treated cells compared to the controls, and there were no differences between the hyperoxic cells and the rIL-10-treated cells. The results are represented as the mean ± SD from three different experiments.

Expression of IL-10 receptors was evaluated by ELISA and qRT-PCR. As shown in Figure2, IL-10 receptor-α and -β protein increased 3.2-fold and 2.4-fold, respectively, after 24 hrs of hyperoxia compared to the controls (Fig.2A and B). Similarly, IL-10 receptor-α and -β mRNA increased 3.3-fold and 1.4-fold, respectively, after exposure to hyperoxia for 24 hrs compared to the controls (Fig.2C and D). Also, marked increases in IL-10 receptor-α and -β protein (Fig.2A and B) and IL-10 receptors mRNA (Fig.2C and D) were presented in the rIL-10-treated hyperoxic cells compared to the controls, but, these increases did not differ from the increases observed in the rIL-10-untreated hyperoxic cells (Fig.2). And IL-10 receptor-α and -β protein did not increase significantly in the rIL-10-treated control cells compared to the rIL-10-untreated control cells (Fig.2A and B), and their genes expression showed the similar pattern to the proteins (Fig.2C and D).

### IL-10 signalling proteins in FATIICs during hyperoxia

**Figure 3:**
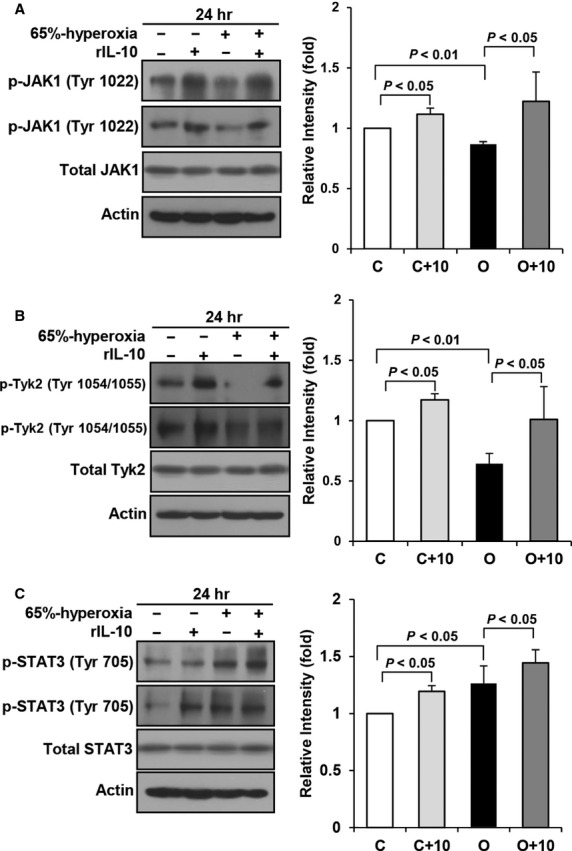
IL-10 signalling proteins in the hyperoxic cells and in the rIL-10-treated cells. E19 foetal alveolar type II cells were exposed to 65%-hyperoxia for 24 hrs. IL-10 signalling proteins were assessed by Western blot using phospho-specific antibodies. During hyperoxia, phosphorylated JAK1 (A), TYK2 (B) were significantly suppressed, and STAT3 (C) significantly enhanced compared to the controls, and they were enhanced in the rIL-10-treated cells compared to the untreated cells (A–C). C, O and 10 mean control, 65%-oxygen and rIL-10, respectively. The results are represented as the mean ± SD from three different experiments.

Next, we examined the IL-10 signalling proteins by Western blot using phospho-specific antibodies under four conditions: control (normoxic), rIL-10-treated control, hyperoxic, and rIL-10-treated hyperoxic cells. As shown in Figure3A and B, phosphorylated JAK1 (p-JAK1) and TYK2 (p-TYK2) were significantly suppressed by 17% and 50% in the hyperoxic cells compared to the control cells and were significantly enhanced 1.6-fold and 2.1-fold, respectively, in the rIL-10-treated hyperoxic cells compared to the rIL-10-untreated hyperoxic cells (Fig.3A and B). In contrast with p-JAK1 and p-TYK2, phosphorylated STAT3 (p-STAT3) was enhanced significantly 1.3-fold in the hyperoxic cells compared to the control cells (Fig.3C) and also increased 1.2-fold in the rIL-10-treated hyperoxic cells compared to the rIL-10-untreated hyperoxic cells (Fig.3C).

### IL-10 signalling proteins in IL-10 receptors-blocked type II cells

**Figure 4:**
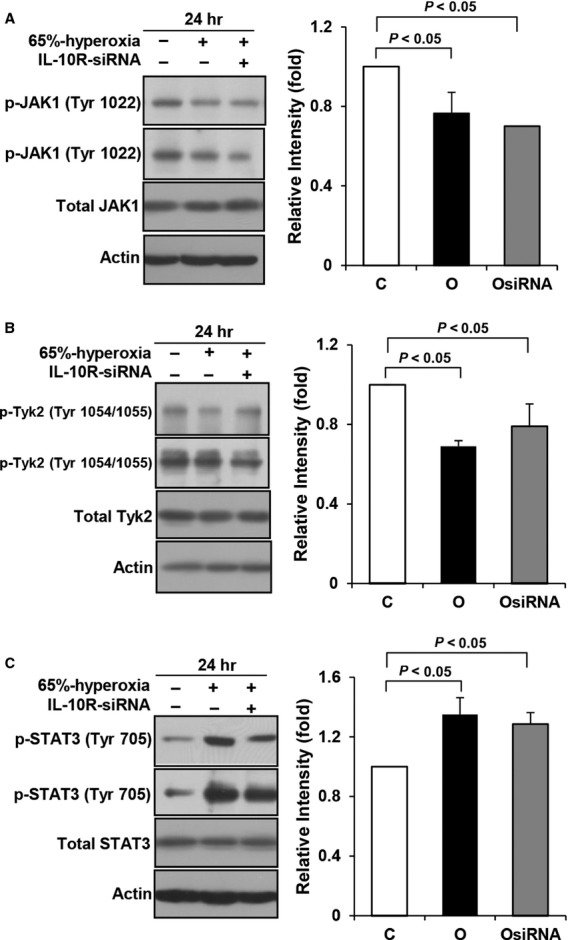
IL-10 signalling proteins in the IL-10 receptors-blocked cells. E19 foetal alveolar type II cells were exposed to 65%-hyperoxia for 24 hrs. IL-10 signalling proteins were assessed by Western blot using phospho-specific antibodies after blocking IL-10 receptors with siRNA. Phosphorylated JAK1 (A) and TYK2 (B) were down-regulated, and phosphorylated STAT3 was enhanced in the hyperoxic cells blocked with IL-10 receptors siRNA, compared to the control cells. C and O mean control and 65%-oxygen, respectively, and OsiRNA and IL-10R-siRNA mean 65%-oxygen with IL-10 receptors’ siRNA and IL-10 receptors with siRNA, respectively. The results are represented as the mean ± SD from three different experiments.

Former results showed that IL-10 signalling proteins (JAK1 and TYK2) and IL-10 receptors followed the opposite direction. Next, to address that this phenomenon is a compensatory response attempting to increase IL-10 or mediated *via* other mechanisms, we evaluated the signalling proteins after blocking IL-10 receptors with siRNA. As a result, p-JAK1 and p-TYK2 decreased in the IL-10 receptors-blocked hyperoxic cells, and these results showed the same pattern with the hyperoxic cells without siRNA for IL-10 receptors (Fig.4A and B). Also, p-STAT3 increased in the IL-10 receptors-blocked hyperoxic cells, which was similar pattern with the IL-10 receptors-unblocked hyperoxic cells (Fig.4C).

### Effect of IL-8 on IL-10 receptors and IL-10 signalling proteins in FATIICs

**Figure 5:**
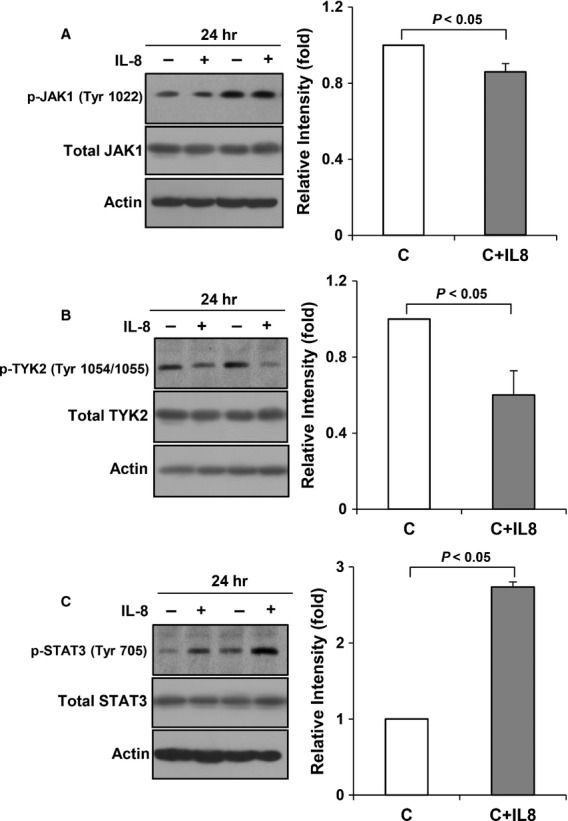
Effect of IL-8 on IL-10 receptors and IL-10 signalling proteins in the absence of hyperoxia. E19 foetal alveolar type II cells were exposed to 65%-hyperoxia for 24 hrs. IL-10 signalling proteins were assessed by Western blot using phospho-specific antibodies. Phosphorylated JAK1 (A) and TYK2 (B) decreased, and phosphorylated STAT3 (C) increased in the IL-8-added cells, compared to the control cells. C means control. The results are represented as the mean ± SD from three different experiments.

**Figure 6:**
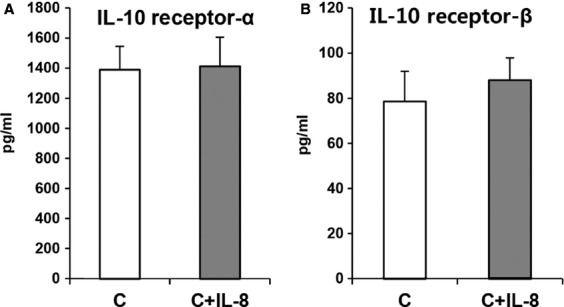
IL-10 receptors protein in the IL-8-added cells in the absence of hyperoxia. E19 foetal alveolar type II cells were exposed to 65%-hyperoxia for 24 hrs. IL-10 receptors protein was assessed by ELISA. IL-10 receptor-α (A) and -β protein (B) did not change in the IL-8-added cells, compared to the control. C means control. The results are represented as the mean ± SD from three different experiments.

Considering the former results overall, the changes of IL-10 signalling proteins may be related to the other mechanism such as increased pro-inflammatory cytokines secondary to hyperoxia. Hence, we evaluated the impact of IL-8 shown previously to be increased by hyperoxia in FATIICs 9 on IL-10 receptors and signalling proteins in the absence of hyperoxia. As shown in Figure5A and B, p-JAK1 and p-TYK2 significantly decreased by 24% and 40%, respectively, in the IL-8-added control cells compared to the control cells without IL-8 (Fig.5A and B), and these phenomena were similar to those observed in the hyperoxic cells (Fig.3A and B). And p-STAT3 increased 2.7-fold in the IL-8-added control cells compared to the controls without IL-8 (Fig.5C), which was the similar response with that shown in the hyperoxic cells (Fig.3C). Taking overall, the IL-8-added control cells had the same response in IL-10 signalling proteins with the hyperoxic cells (Figs3 and 5). In contrast with the IL-10 signalling proteins, IL-10 receptors did not change in the IL-8-added control cells as compared to the control cells without IL-8 (Fig.6A and B).

## Discussion

The main findings of the present study are as follows. Interleukin-10 production decreased in an oxygen-concentration-dependent manner in FATIICs. Interleukin-10 receptors were increasingly expressed in FATIICs during hyperoxia. However, IL-10’s signalling proteins including JAK1 and TKY2 were suppressed, and STAT3 was enhanced in FATIICs during hyperoxia. In addition, pre-treatment of FATIICs with rIL-10 prior to hyperoxia exposure increased p-JAK1, p-TYK2 and p-STAT3. Interleukin-10 receptors-blocked hyperoxic cells showed the same pattern in IL-10 signalling proteins with the hyperoxic cells, and IL-8-added control cells showed the same pattern in signalling proteins with the hyperoxic cells.

We focused on FATIICs in this study. The reason for this was that alveolar type II cells play central roles as barrier cells of the alveolar mucosa and as stem cells for the restoration of alveolar epithelium following acute lung injury 26; additionally, alveolar type II cells express innate immune genes 27.

In a recent study, the selection of 65%-oxygen was based on a previous finding that exposure to 65%-hyperoxia in newborn mice resulted in impaired lung architecture in adult mice 28, and 60%-hyperoxia exposed to newborn mice promoted alveolar simplification in adult mice 29. In addition, our previous investigations showed that 65%-hyperoxia induced alveolar type II cell injury in foetal rats aged on E19 and pre-incubation of rIL-10 protected FATIICs from injury secondary to 65%-hyperoxia 9,21.

Recently, there has been growing attention regarding the protective potency of exogenous IL-10 in preventing BPD in preterm infants 13,30. Li *et al*. reported that rIL-10 significantly inhibited IL-6 and TNFα production in macrophages from tracheobronchial aspirate fluid from newborn infants stimulated by lipopolysaccharide 15. Additionally, we previously demonstrated that pre-treatment of FATIICs with rIL-10 prior to hyperoxia exposure augmented IL-8 production and cellular necrosis in FATIICs and increased type II cell proliferation compared to the untreated cells 9.

Our results concerning IL-10 production from FATIICs at different concentrations of hyperoxia showed that IL-10 release decreased with respect to hyperoxia concentration. These results suggest that the capability of IL-10 in the neonatal lungs is limited inversely with hyperoxia concentration and that lack of IL-10 may play an important role in the genesis of lung injury in preterm infants.

Interleukin-10 exerts its biological effects on cells by interacting with a specific cell surface receptor 31. The IL-10 receptor is composed of two distinct subunits, α and β 20, and both subunits are known to have roles in signal transduction events 32,33. Interleukin-10 receptor-α plays the dominant role in mediating high-affinity ligand binding and signal transduction events 32, and IL-10 receptor-β is known as the orphan receptor, serving an accessory chain essential for the active IL-10 receptor complex; it initiates IL-10 induced signal transduction events 33. Previous reports that support the role of IL-10 receptor-β in the IL-10 receptor complex have shown that IL-10 receptor-β-/- mice developed severe enterocolitis, and the cells from these mice were unresponsive to IL-10 31,34,35. Hence, these two receptor complexes are necessary to mediate the immunosuppressive signals of IL-10, thus to inhibit the synthesis of pro-inflammatory cytokines. In the present study, IL-10 receptors and their genes expression were significantly enhanced during hyperoxia in FATIICs compared to the control samples, but did not increase further in the rIL-10-treated hyperoxic cells as compared to the rIL-10-untreated hyperoxic cells. Similarly, IL-10 receptors did not increase in the rIL-10-treated control cells compared to the control cells without rIL-10. These overall findings suggest that the IL-10 receptors significantly increased in FATIICs during hyperoxia, which may not be ligand-specific, and the exogenous IL-10 may initiate its signalling through binding to the expressed IL-10 receptor-α and -β related to the activation of JAK1 and TKY2, respectively in FATIICs during hyperoxia 18.

Our observation presented that 65%-hyperoxia down-regulates JAK1 and TYK2 and up-regulates STAT3, and pre-treatment of rIL-10 increased the activation of JAK1, TYK2 and STAT3. And the activation of IL-10 signalling proteins after blocking the IL-10 receptors with siRNA had the same response with the hyperoxic cells. These results suggest that IL-10 receptors do not seem to play a significant role in the downstream IL-10 signalling proteins, and the increased IL-10 receptors may be compensatory response attempting to increase IL-10 during hyperoxia.

Our observations showed that only p-STAT3 increased in FATIICs exposed to hyperoxia, unlike to p-JAK1 and p-TYK2. Based on previous evidence showing that the signalling protein, STAT3 of the IL-10 pathway is activated upon stimulation of IL-8 in dermal microvascular endothelial cells in human 36,37 and in halothane treated IL-10 KO mice, we investigated the impact of IL-8 shown previously by the authors to be increased in FATIICs during hyperoxia, on IL-10 receptors and the signalling proteins in the absence of hyperoxia. As a result, the present study showed that IL-10 receptors did not change in the IL-8-added control cells, compared to the control cells without IL-8, but JAK1 and TYK2 decreased, and STAT3 increased in the IL-8-added control cells. These responses of IL-10 signalling proteins in the IL-8-added control cells were the same pattern with the hyperoxic cells. These findings suggest that IL-8 may play an important role to attenuate IL-10 signalling by blocking JAK/Tyrosine kinase activity.

In summary, the present study is the first experimental *in-vitro* study to investigate the expression of IL-10 receptors and the activation of IL-10 signalling proteins, as well as the effect of rIL-10 on IL-10 receptors and IL-10 signalling proteins in FATIICs exposed to hyperoxia and suggest that exogenous IL-10 could manage to exert its anti-inflammatory property in FATIICs during hyperoxia through binding to the IL-10 receptors and improving the activation of IL-10 signalling proteins, JAK1 and TYK2.
